# Rapid Determination of 21 Chinese Domestically Registered Pesticides in Ginseng Using Cleanup Based on Zirconium-Oxide-Modified Silica and Ultrahigh-Performance Liquid Chromatography-Tandem Mass Spectrometry

**DOI:** 10.1155/2021/5516563

**Published:** 2021-08-13

**Authors:** Zhongbei Zhang, Zhou Lu, Nan Fang, Zhiguang Hou, Weiming Ren, Yueru Li, Zhongbin Lu

**Affiliations:** ^1^College of Plant Protection, Jilin Agricultural University, Changchun 130118, Jilin, China; ^2^Laboratory of Quality & Safety Risk Assessment for Ginseng and Antler Products, Jilin Agricultural University, Changchun 130118, Jilin, China

## Abstract

In this study, an analytical method was developed for the rapid determination of 21 pesticides used in ginseng cultivation. All pesticides covered by this method have been registered by 2020 in China for use on ginseng. The extracts were cleaned up using zirconium-oxide-modified silica (Z-Sep) and primary secondary amine (PSA). The combination of Z-Sep and PSA provided good recovery for all analytes and the cleanest matrix background out of a number of PSA-based sorbent combinations, as indicated by high-performance liquid chromatography (HPLC) and gas chromatography (GC). Instrumental analysis was completed in 5 min using the ultrahigh-performance liquid chromatography-tandem mass spectrometry (UHPLC-MS/MS). The linearity (*r* > 0.99) for all analytes was satisfactory over the calibration range of 0.002–0.1 *μ*g mL^−1^. Intraday recoveries (*n* = 5) at ginseng-spiked levels of 0.02, 0.05, 0.1, and 1 mg kg^−1^ ranged between 72% and 119%, with the corresponding relative standard deviations (RSDs), were less than 19%, while the interday recoveries (*n* = 15) ranged between 77% and 103%, and RSDs were less than 22%. Limits of quantitation (LOQs) ranged between 0.02 and 0.05 mg kg^−1^ for all 21 pesticides. This is a seminal study using Z-Sep for the efficient cleanup of ginseng samples, and it could present a practical method for future monitoring of pesticide residues in ginseng produced in China.

## 1. Introduction

Ginseng refers to the dried root of *Panax ginseng* C. A. Meyer, which belongs to the Araliaceae family, and is important in traditional Chinese medicine. The active components in ginseng, ginsenosides, exhibit antioxidant and antitumor properties [1]. Ginseng is also rich in polysaccharides and amino acids [2], making it a valuable dietary supplement. Like other crops, ginseng plants are prone to diseases such as blight, gray mold, and black spots as well as underground pests such as wire- and cutworms. Thus, it is necessary to apply pesticides during the cultivation of ginseng [3]. *Panax ginseng* is a perennial plant and needs to grow for at least three years before the roots are ready for harvesting and further commercialization. Agricultural pesticides may remain in the soil for a long time, and the roots of perennial plants are at risk of repeated contamination.

China is the second-largest exporter of ginseng worldwide [4]. Over one thousand tons of ginseng are exported from China to other countries. Therefore, ensuring that ginseng produced in China is of high quality and safe for human consumption is crucial for the global market. As of 2020, there are 32 active ingredients in pesticides registered for the control of various pathogens and pests on ginseng [5]; details of these compounds and their uses are shown in Supplementary Table S1. Previous multiresidue studies conducted using ginseng have revealed residues containing many obsolete pesticides, such as organochlorine and organophosphorus [6–11]. These compounds are usually analyzed by gas chromatography (GC), which is time-consuming and requires the analytes to be volatile. Conversely, high-performance liquid chromatography (HPLC) and ultrahigh-performance liquid chromatography (UHPLC) methods provide more rapid analyses and broader detection spectra without the requirement of analyte volatility; thus, they have been widely applied in the past decade [12–17]. Throughout all of our reviewed references, two studies each determined two hundred more pesticides using either GC or liquid chromatography (LC) approaches are very representative. Hayward et al. [18] analyzed 310 pesticides and relevant metabolites in ginseng using gas chromatography-tandem mass spectrometry (GC-MS/MS), and Chen et al. [14] developed a method for the determination of 236 pesticides in ginseng using high-performance liquid chromatography coupled with tandem mass spectrometry (HPLC-MS/MS). Nevertheless, these studies only covered 10 and 14 of the pesticides, respectively, currently registered for use on ginseng in China.

Food samples must undergo various preparation procedures before being suitable for instrumental analysis [19–21]. The quick, easy, cheap, effective, rugged, and safe (QuEChERS) method is a modern technique developed to fulfill this purpose rapidly [22]. QuEChERS uses acetonitrile (ACN) salt-out extraction, followed by cleanup with primary secondary amine (PSA), octadecyl silica (C_18_), and graphitized carbon black (GCB) to provide accurate measurements. Recently, a number of novel materials have been introduced to improve cleanup efficiency during QuEChERS treatments. Zirconium-oxide-modified silica (Z-Sep) could be a promising substitute for traditional cleanup sorbents. Residue analyses in earlier studies have confirmed that Z-Sep efficiently removes lipids from various fat-rich food matrices [23–25]. Others have found that Z-Sep decreases the amounts of coextractives and lowers the matrix effects in food matrices that are not fat-rich, compared to some traditional sorbents [26–28].

In this study, we developed and validated a rapid multiresidue method for the simultaneous determination of 21 pesticides in ginseng using the ultrahigh-performance liquid chromatography coupled with tandem mass spectrometry (UHPLC-MS/MS). All the compounds that we tested are registered pesticides for use in ginseng cultivation in China. The sample was cleaned up using Z-Sep following QuEChERS ACN salt-out extraction; UHPLC-MS/MS analysis took only 5 min. This method provides efficient sample purification and is suitable for the rapid determination of pesticide residues in ginseng produced in China.

## 2. Materials and Methods

### 2.1. Chemicals and Materials

Analytical standards of azoxystrobin (99.2%), carbendazim (98.6%), cymoxanil (99.0%), cyprodinil (99.9%), diethofencarb (99.6%), difenoconazole (99.9%), dimethomorph (98.7%), fluazinam (99.9%), fludioxonil (99.8%), flumorph (96.5%), fluopyram (99.0%), flusilazole (98.0%), kresoxim-methyl (97.8%), mandipropamid (99.4%), metalaxyl (99.9%), propamocarb (97.1%), propiconazole (99.0%), pyrimethanil (99.9%), thiamethoxam (99.6%), and trifloxystrobin (99.4%) were provided by Dr. Ehrenstorfer GmbH (Augsburg, Germany). Analytical standard of pyraoxystrobin (95.0%) was obtained from Alta Scientific Ltd. (Tianjin, China). ACN of HPLC grade was provided by DiKMA Technologies, Inc. (Beijing, China). Water used in this study was prepared by a Milli-Q water purification system (Burlington, USA). Ammonium formate (≥99.995%) was purchased from Sigma-Aldrich, Inc. (St. Louis, USA). Anhydrous MgSO_4_ and NaCl were bought from Agilent Technologies (Santa Clara, USA). Z-Sep (22 *μ*m) was purchased from Sigma-Aldrich, Inc. (St. Louis, USA). Seventy ginseng samples were purchased from commercial sources, and the blank matrices were obtained from the standardized ginseng cultivation base. The pesticides involved in the method were never applied during the cultivation of the ginseng.

A stock solution of 21 pesticides with a concentration of 100 *μ*g mL^−1^ was prepared by dissolving 5 mg of each pesticide standard in 50 mL of ACN followed by ultrasonication for 5 min. The stock solution was kept in an amber vial and stored in a freezer at −30°C. Stock solutions of 10 *μ*g mL^−1^ for mixed pesticides were prepared in ACN. An external calibration standard curve for matrix-matched quantitation was prepared by diluting the stock solution with blank ginseng extract provided using the developed method, at the concentrations of 0.002, 0.005, 0.02, 0.05, and 0.1 *μ*g mL^−1^. All standards were prepared immediately prior to use.

### 2.2. Sample Preparation

The ginseng sample was ground to a fine powder using an electric grinder (Linda Machinery Co. Ltd., China) and passed through a 0.355 mm sieve. For pretreatment, samples (2.0 g) were weighed and placed in a 50 mL polypropylene centrifuge tube. After adding 10.0 mL of water, the tube was vortexed by an IKA vortex 2 (IKA Works, Guangzhou, China) at 2,500 rpm for 20 seconds. It was then allowed to stand for 15 min to hydrate. Then, 10.0 mL ACN was added, and the tube was capped and vortexed at the same speed for 2 min. Thereafter, 4.0 g of anhydrous MgSO_4_ and 1.0 g of NaCl were added. The tube was vortexed for 1 min followed by centrifugation at 4,500 rpm for 5 min, and 1.0 mL of the resulting supernatant was transferred to a 2.0 mL centrifuge tube containing 50 mg PSA, 50 mg Z-Sep, and 150 mg anhydrous MgSO_4_. This mixture was vortexed for 10 seconds followed by centrifugation at 4,500 rpm for 5 min. The supernatant was then passed through a 0.22 *μ*m nylon syringe filter into the injection vial for subsequent UHPLC-MS/MS analysis.

### 2.3. Instrumentation

#### 2.3.1. Analysis of 21 Pesticides by UHPLC-MS/MS

Nexera X2 UHPLC (Shimadzu Corporation, Japan) coupled with a QTRAP4500 MS/MS (AB Sciex Pte. Ltd., USA) was used to detect 21 pesticides in the ginseng samples. The UHPLC system was equipped with a ZORBAX Eclipse C_18_ column (2.1 × 50 mm, 1.8 *μ*m; Agilent Technologies, USA), which was held at 40°C in a column oven. The LC mobile phase consisted of phase A (5 mmol L^−1^ ammonium in water) and phase B (ACN). The flow rate was 0.4 mL min^−1^. The gradient elution started at 40% B, was increased linearly to 95% B for 3.0 min, kept constant for 1.0 min, then switched back to 40% B over 0.1 min, and eventually allowed to equilibrate for 0.9 min. The injection volume was 2 *μ*L.

Electrospray ionization with tandem mass spectrometry (ESI-MS/MS) was performed using multiple reaction monitoring (MRM) in both positive and negative modes. The ion source conditions were as follows: ion spray voltage, 5,500 V (ESI+)/−4,500 V (ESI−); source temperature, 550°C; curtain gas pressure, 35 psi; ion spray gas pressure, 45 psi; and auxiliary heating gas pressure, 45 psi. To identify characteristic ion transitions of each pesticide, the standard solution containing each analyte at 1.0 *μ*g mL^−1^ was continuously infused to the MS/MS at a speed of 20 *μ*L min^−1^ using a syringe pump. Two characteristic ion transitions were selected for each analyte, with the strongest intensities serving for quantitation. The dwell time of each ion transition in MS was set to 2 ms. The declustering potential (DP), collision energy (CE), and collision cell exit potential (CXP) were optimized for each individual analyte by running a ramp over a range of each parameter and selecting the value that yielded the strongest ion intensity. The MS detection information for the 21 pesticides is detailed in Table 1.

#### 2.3.2. Investigation of Matrix Background by HPLC-UVD and GC-FID

The matrix background under different cleanup treatments was compared using HPLC and GC. The HPLC system was a Shimadzu LC-20A coupled with an ultraviolet detector (UVD). A Thermo BDS C_18_ column (250 × 4.6 mm; 5 *μ*m) was equipped, and the column oven was set at 40°C. The mobile phases comprised phase A (0.1% acetic acid in water) and phase B (ACN). An isocratic elution of 65%B flowed at 1.0 mL min^−1^ for 15 min. The injection volume was 10 *μ*L, and the absorption wavelength of the UVD was set to 254 nm.

GC was performed using a Shimadzu GC-2010 Plus coupled with a flame ionization detector (FID). The system was equipped with an Agilent DB-WAX column (polyethylene glycol; 30 m × 0.32 mm; 0.25 *μ*m). The injection port and detector temperatures were 250°C and 285°C, respectively. Ultrapure nitrogen (>99.999%) served as the carrier gas and flowed at a rate of 1.0 mL min^−1^. The oven temperature was initially held at 60°C for 1 min, increased to 140°C at a rate of 10°C min^−1^ and held constant for 5 min, increased to 200°C at 20°C min^−1^ and held constant for 10 min, and then finally increased to 280°C at 20°C min^−1^ and held constant for 10 min. The flow rates of air and hydrogen used for ignition were set to 400 mL min^−1^ and 40 mL min^−1^, respectively. The scan rate of the FID was 40 ms. The splitless injection mode was employed, and the injection volume was 1 *μ*L.

### 2.4. Method Validation

Quantitative analysis of the 21 pesticides was achieved using a 5-point (0.002, 0.005, 0.02, 0.05, and 0.1 *μ*g mL^−1^) matrix-matched calibration curve. Recovery measurements were conducted by adding 100 *μ*L of 0.2, 0.5, 1.0, and 10 *μ*g mL^−1^ pesticide standard mixtures to 2.0 g ± 0.05 of blank matrices to achieve spiked levels of 0.02, 0.05, 0.1, and 1 mg kg^−1^, respectively. Because 1 mg kg^−1^ exceeded the range of the matrix-matched calibration curve, we used blank ginseng extract to dilute the sample extract spiked to this concentration to an acceptable concentration before detection. The method was validated using recoveries (intraday and interday), and the corresponding relative standard deviations (RSDs) returned after spiking blank ginseng samples at the abovementioned concentrations.

According to SANTE guidelines, the limit of quantitation (LOQ) is the lowest spiked level of the validation meeting method performance acceptability criteria [29]. Matrix effects (ME) of each analyte in the ginseng matrix were determined using the following equation:

ME = (slope of matrix-matched standard curve/slope of solvent calibration curve − 1) × 100% [30].

### 2.5. Method Application to Real Samples

Seventy ginseng samples were collected from processing firms and traditional Chinese medicine pharmacies in Jilin Province, China's major ginseng production area. All samples were pretreated using the developed method to screen for target pesticide residue content.

## 3. Results and Discussion

### 3.1. Pesticide Detection Spectrum

As of 2020, 32 pesticides have been registered in China for use during ginseng cultivation (Supplementary Table S1), and 21 of them were covered in the detection spectrum of this method. Other registered pesticides that could not be incorporated into this method include the biological fungicides *Bacillus subtilis*, *Paenibacillus polymyxa*, and *Trichoderma harzianum*, which are exempt from the global maximum residue limit (MRL) regulations. Inorganic fungicides such as copper hydroxide and copper oxychloride are not LC-MS-amenable and need to be determined by atomic absorption spectroscopy or the colorimetric method [31]. The nucleoside antibiotic polyoxin can only be detected using pure water as the extraction solvent, which is not compatible with the ACN salt-out extraction we used [32, 33]. The dithiocarbamate fungicide mancozeb is not LC-MS-amenable and regularly determined by reaction with acid to form carbon disulfide, which is measured by GC-FPD [31]. Heteroaromatic fungicide hymexazol, dicarboximide fungicide iprodione, and plant growth regulator gibberellic acid showed low response on the LC-MS we employed, and their detection did not reach the desirable LOQs meeting MRLs, and thus, they were not included in this method.

### 3.2. Sample Extraction and Cleanup

**Figure 1:**
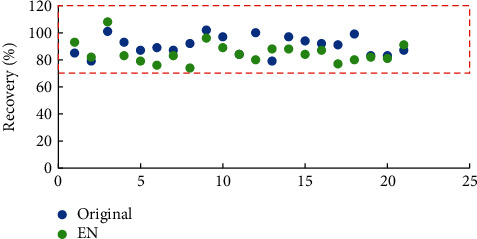
Recoveries (*n* = 5) of 21 pesticides from ginseng samples extracted by different QuEChERS methods.

In our study, the original and citrate-buffered QuEChERS methods were used for extraction of spiked ginseng samples, and the results were compared to identify which method provided satisfactory recoveries.  Figure 1 shows that the recoveries of 21 pesticides from samples extracted by both methods were within the acceptable range (70–120%) defined by the SANTE guidelines [29]. In the case of 15 pesticides, better recoveries were provided by original extraction. We deduced that this was because not all pesticides tested were pH-sensitive, and more salts added in the citrate-buffered method may have hindered the full dispersion of the sample in the extraction solution. As such, the original QuEChERS extraction method was adopted.

**Figure 2:**
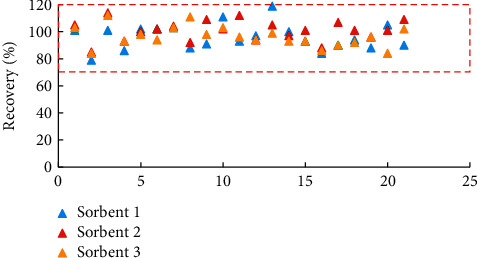
Recoveries (*n* = 5) of 21 pesticides from samples cleaned up by different sorbents (sorbent 1, 50 mg PSA + 50 mg C_18_; sorbent 2, 50 mg PSA + 50 mg FLS; and sorbent 3, 50 mg PSA + 50 mg Z-Sep).

In the present study, different sorbent combinations comprised PSA, C_18_, and Florisil (FLS) that were combined with the novel Z-Sep as follows: (a) 50 mg PSA plus 50 mg C_18_, (b) 50 mg PSA plus 50 mg FLS, and (c) 50 mg PSA plus 50 mg Z-Sep. Ginseng contains only minor pigments, and its crude ACN extract only showed a light yellowish color (Supplementary Figure S1); thus, GCB, a sorbent commonly used for pigment removal, was not considered in the method development. Recoveries from the extracts purified by a, *b*, and *c* sorbent combinations were in the ranges of 79–119%, 85–114%, and 84–112%, respectively (Figure 2), with no significant difference (RSD < 15%). The results indicated that all sorbent combinations provided satisfactory recoveries with no undesired retention.

**Figure 3:**
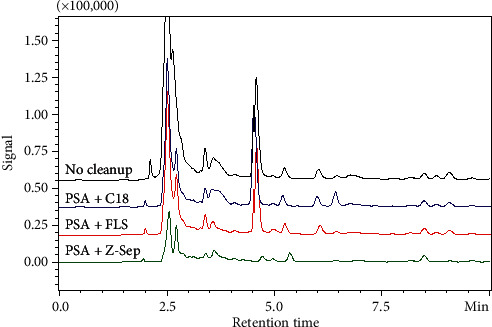
Matrix background of ginseng samples purified using different strategies indicated by HPLC-UVD.

**Figure 4:**
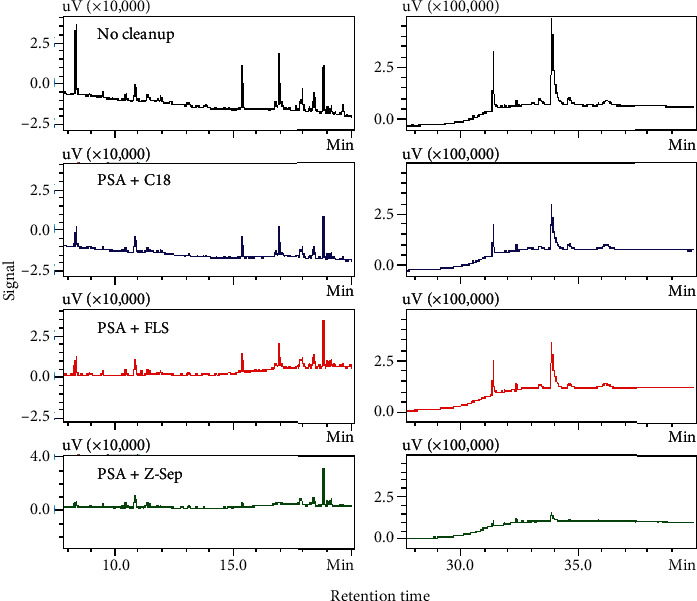
Matrix background of ginseng samples purified using different strategies indicated by GC-FID.

The aim of cleanup is to achieve good recovery while also removing coextractives from the sample. As the ginseng matrix contains both LC-amenable, such as ginsenosides and polysaccharides, and GC-amenable components [34], the use of LC and GC together would provide a thorough profile of coextractives in ginseng before and after the cleanup.  Figure 3 shows the matrix backgrounds of samples under different cleanup treatments. The coextractive peaks of crude ginseng extract mainly eluted between 2.0 and 3.0 min, with minor peaks appearing at 4.7 min. Sorbent combinations a and *b* both removed large portions of coextractives eluted in the early period but not those eluted at 4.7 min. Sorbent combination *c* further reduced peaks between 2.0 and 3.0 min and eliminated the coextractive eluted at 4.7 min. Conversely, matrix background investigation by GC (Figure 4) indicated that volatile components in the samples mainly eluted between 8–20 min and 30–37 min. Sorbent combination *c* provided a cleaner background than the other two treatments.

Previous studies have focused on the lipid-removal capability of Z-Sep in fatty samples [23–25]. Unlike C_18_, which uses its long alkyl chain to retain lipid compounds, Z-Sep utilizes empty d-orbitals on zirconium atoms. These act as electron acceptors [35] and adsorb fatty acids, as well as a wide range of compounds such as amino acids and phenols in the matrices. A schematic diagram of the adsorption mechanism of Z-Sep during sample cleanup is shown in Supplementary Figure S2.

Compared with other sorbents, the combination of 50 mg PSA and 50 mg Z-Sep yielded satisfactory recoveries for all analytes and reduced the matrix background to the minimum indicated by HPLC and GC; thus, it was chosen for the cleanup of ginseng samples in our developed method.

### 3.3. Method Validation

Multiple reaction monitoring (MRM) chromatograms of the 21 pesticides are shown in Supplementary Figure S3. Double peaks were observed for dimethomorph because the standard substance of this compound contains two isomers (E and Z), which can be partly or completely resolved on a C_18_ column depending on the chromatographic conditions [36, 37]. The European Commission specifies that the sum of these two isomers is the residue definition of dimethomorph [38]. Therefore, quantitation of dimethomorph was achieved by integrating this double-peak signal.

The calibration information, ME, and LOQs of the 21 pesticides are shown in Table 2. The linearity was good (*r* > 0.99) for all pesticides in the calibration range of 0.002 to 0.1 *μ*g mL^−1^. LOQs for cymoxanil, diethofencarb, fluazinam, fludioxonil, kresoxim-methyl, propamocarb, and thiamethoxam were defined at 0.05 *μ*g mL^−1^, whereas, for others, it was defined at 0.02 *μ*g mL^−1^. The LOQs of the 21 pesticides were lower than or equal to the MRLs regulated by China and the European Union (EU). At different ginseng spike levels, the intraday recovery range for all pesticides was 72–119% (*n* = 5 and RSDs < 19%), and the interday recovery range was 77–103% (*n* = 15 and RSDs < 22%). The detailed recoveries and RSDs are listed in Supplementary Table S2.

### 3.4. Real Sample Analysis

Seventy real samples of ginseng were analyzed using the developed method for screening of the target pesticides (Table 3), and each sample was only detected once. Residues (>LOQs) of azoxystrobin, difenoconazole, dimethomorph, and pyrimethanil were detected at frequencies ranging between 2.9% and 7.1%. All pesticides detected were lower than the corresponding MRLs regulated by China and the EU. Dimethomorph and azoxystrobin were also detected in the study by Chen et al. [14]. Fungicide use in ginseng cultivation is clearly widespread; therefore, attention should be paid to fungicide detection in ginseng and expanding the list of detected fungicides.

## 4. Concluding Remarks

In this study, an analytical method was developed for the simultaneous determination of 21 pesticides in ginseng. All pesticides covered by this method have been currently registered for use on ginseng in China. Ginseng samples were extracted using the QuEChERS ACN salt-out extraction methodology followed by dispersive solid-phase extraction (d-SPE) cleanup using Z-Sep and PSA. Z-Sep was first used for purifying ginseng matrix and provided the best cleanup efficiency compared to other PSA-based sorbents. Instrumental analysis was completed rapidly by UHPLC-MS/MS in 5 min. Azoxystrobin, difenoconazole, dimethomorph, and pyrimethanil residues were detected (>LOQs) in real samples of ginseng, with all contents lower than their corresponding MRLs regulated by China and EU. This study provides a practical method for monitoring pesticide residues in ginseng produced in China, which could be crucial given its position as a major exporter of ginseng globally.

## Figures and Tables

**Table 1:** MS/MS detection information of 21 pesticides.

Compound	Ionization mode	RT (min)	Ion transition	Ion ratio	DP (V)	CE (V)	CXP (V)
Azoxystrobin	ESI+	2.08	404.1 > 372.2^a^; 404.1 > 344.2	0.41	50	20; 34	10; 13
Carbendazim	ESI+	0.68	192.0 > 160.1^a^; 192.0 > 132.1	0.27	55	25; 41	7; 5
Cymoxanil	ESI+	0.98	199.0 > 128.1^a^; 199.0 > 111.1	2.60	45	13; 25	7; 7
Cyprodinil	ESI+	2.42	226.1 > 108.1; 226.1 > 93.1^a^	0.62	88	34; 50	9; 7
Diethofencarb	ESI+	2.02	268.1 > 226.2^a^; 268.1 > 198.0	0.35	57	14; 22	9; 7
Difenoconazole	ESI+	2.50	406.0 > 337.0; 406.0 > 251.0^a^	0.25	75	25; 34	12; 10
Dimethomorph	ESI+	1.87	388.1 > 301.1^a^; 388.1 > 165.2	0.33	110	29; 42	12; 10
Fluazinam	ESI−	2.78	463.0 > 415.9^a^; 463.0 > 397.8	0.55	−114	−28; −25	−17; −17
Fludioxonil	ESI−	1.97	247.0 > 180.0; 247.0 > 126.0^a^	1.78	−80	−40; −41	−12; −10
Flumorph	ESI+	1.61	372.2 > 285.1^a^; 372.2 > 165.1	0.50	100	29; 41	11; 6
Fluopyram	ESI+	2.22	397.1 > 208.1^a^; 397.1 > 173.1	1.24	84	30; 40	8; 7
Flusilazole	ESI+	2.20	316.1 > 247.1^a^; 316.1 > 165.1	0.51	100	25; 37	9; 6
Kresoxim-methyl	ESI+	2.50	314.1 > 234.9^a^; 314.1 > 222.1	1.30	53	22; 20	10; 10
Mandipropamid	ESI+	2.11	412.1 > 356.1; 412.1 > 328.1^a^	0.11	94	14; 20	14; 12
Metalaxyl	ESI+	1.59	280.2 > 220.2; 280.2 > 192.1^a^	1.07	60	19; 24	9; 8
Propamocarb	ESI+	0.35	189.1 > 145.2; 189.1 > 102.1^a^	0.42	50	18; 24	6; 7
Propiconazole	ESI+	2.36	342.0 > 204.9; 342.0 > 159.0^a^	0.09	23	26; 39	10; 9
Pyraoxystrobin	ESI+	2.55	413.1 > 204.9; 413.1 > 145.2^a^	1.82	40	16; 35	6; 5
Pyrimethanil	ESI+	1.94	200.0 > 168.1; 200.0 > 107.0^a^	0.36	84	41; 32	6; 7
Thiamethoxam	ESI+	0.55	292.0 > 211.2^a^; 292.0 > 132.1	1.22	55	19; 30	8; 9
Trifloxystrobin	ESI+	2.83	409.1 > 206.1; 409.1 > 186.0^a^	1.00	82	19; 24	7; 7

^a^For use of quantitation, RT: retention time, DP: declustering potential, CE: collision energy, CXP: collision cell exit potential, ESI+: positive mode of the electrospray ionization source, and ESI−: negative mode of the electrospray ionization source.

**Table 2:** Calibration information, matrix effects (ME), limits of quantitation (LOQs), and maximum residue limits (MRLs) of 21 pesticides in ginseng matrix.

Compound	Equation	*r*	ME (%)	LOQ (mg kg^−1^)	MRL (mg kg^−1^)	
					EU	China
Azoxystrobin	*y* = 122905*x* + 35549^*∗*^	0.9979	10	0.02	0.3^a^	1^a^
	*y* = 135490*x* + 70269^*∗∗*^	0.9972				
Carbendazim	*y* = 126295*x* – 3796^*∗*^	0.9969	−28	0.02	0.1^a^	N.R.
	*y* = 91175*x* + 125830^*∗∗*^	0.9962				
Cymoxanil	*y* = 4741*x* + 5359^*∗*^	0.9970	−1	0.05	0.1^a^	N.R.
	*y* = 4704*x* + 8772^*∗∗*^	0.9932				
Cyprodinil	*y* = 48499*x* − 2204^*∗*^	0.9968	−31	0.02	1.5^a^	N.R.
	*y* = 33292*x* + 32686^*∗∗*^	0.9979				
Diethofencarb	*y* = 4691*x* + 6462^*∗*^	0.9943	38	0.05	0.05^a^	N.R.
	*y* = 6467*x* + 2852^*∗∗*^	0.9944				
Difenoconazole	*y* = 26113*x* + 14194^*∗*^	0.9945	21	0.02	20^a^	0.5^a^
	*y* = 31479*x* + 3037^*∗∗*^	0.9914				
Dimethomorph	*y* = 30402*x* – 2996^*∗*^	0.9987	−2	0.02	0.05^a^	N.R.
	*y* = 29790*x* + 30642^*∗∗*^	0.9951				
Fluazinam	*y* = 4004*x* – 2173^*∗*^	0.9985	−18	0.05	3^a^	N.R.
	*y* = 3287*x* + 1314^*∗∗*^	0.9991				
Fludioxonil	*y* = 3978*x* – 534^*∗*^	0.9993	48	0.05	4^a^	N.R.
	*y* = 5875*x* – 1323^*∗∗*^	0.9919				
Flumorph	*y* = 44444*x* – 4398^*∗*^	0.9959	−6	0.02	N.R.	N.R.
	*y* = 41813*x* + 5597^*∗∗*^	0.9956				
Fluopyram	*y* = 42750*x* + 28644^*∗*^	0.9967	−7	0.02	2.5^a^	N.R.
	*y* = 39881*x* + 116^*∗∗*^	0.9984				
Flusilazole	*y* = 31210*x* + 193^*∗*^	0.9981	−13	0.02	0.05^a^	N.R.
	*y* = 27086*x* + 20576^*∗∗*^	0.9911				
Kresoxim-methyl	*y* = 2996*x* + 7992^*∗*^	0.9965	−28	0.05	0.05^a^	0.1^a^
	*y* = 2145*x* + 1515^*∗∗*^	0.9978				
Mandipropamid	*y* = 31836*x* + 17122^*∗*^	0.9947	7	0.02	0.05^a^	N.R.
	*y* = 34092*x* + 4007^*∗∗*^	0.9989				
Metalaxyl	*y* = 76772 + 32284^*∗*^	0.9995	−4	0.02	0.05^a^	N.R.
	*y* = 73816*x* + 21041^*∗∗*^	0.9990				
Propamocarb	*y* = 17791*x* + 422^*∗*^	0.9943	−60	0.05	0.05^a^	N.R.
	*y* = 7060*x* + 422^*∗∗*^	0.9943				
Propiconazole	*y* = 11509*x* – 1167^*∗*^	0.9994	38	0.02	0.05^a^	0.1^a^
	*y* = 15842*x* – 4597^*∗∗*^	0.9957				
Pyraoxystrobin	*y* = 157075*x* + 14500^*∗*^	0.9905	−14	0.02	N.R.	N.R.
	*y* = 134897*x* + 30881^*∗∗*^	0.9924				
Pyrimethanil	*y* = 30413 + 14032^*∗*^	0.9996	−32	0.02	1.5^a^	1.5^a^
	*y* = 20662*x* + 15146^*∗∗*^	0.9987				
Thiamethoxam	*y* = 3549*x* + 2249^*∗*^	0.9916	35	0.05	0.05^a^	N.R.
	*y* = 4781*x* + 2518^*∗∗*^	0.9950				
Trifloxystrobin	*y* = 21050*x* + 21118^*∗*^	0.9916	−12	0.02	0.05^a^	N.R.
	*y* = 18442*x* + 12744^*∗∗*^	0.9987				

^a^Residue definition of the pesticide is specified to be the parent compound only. N.R.: not regulated, ^*∗*^linear equations of pure standard calibration curves of pesticides, and ^*∗∗*^linear equations of matched-matrix standard calibration curves of pesticides.

**Table 3:** Pesticide detected in real samples of ginseng (*n* = 70) using the developed method.

Compound	Frequency (%)	Detection range (mg kg^−1^)
Azoxystrobin	4.3	0.020–0.101
Difenoconazole	7.1	0.024–0.044
Dimethomorph	4.3	0.044–0.048
Pyrimethanil	2.9	0.024–0.359

## Data Availability

The data used to support the findings of this study are included within the article.

## Supplementary Materials

Supplementary Materials With the manuscript, there are some supplementary materials. These supplementary figures and tables are quoted in the corresponding positions and can be viewed at any time.
