# Supplementary material for: Rapid Determination of 21 Chinese Domestically Registered Pesticides in Ginseng Using Cleanup Based on Zirconium-Oxide-Modified Silica and Ultrahigh-Performance Liquid Chromatography-Tandem Mass Spectrometry
Source: J Anal Methods Chem. 2021 Aug 13;2021:5516563. doi: 10.1155/2021/5516563 (PMC8378960; doi:10.1155/2021/5516563)
Supplement: Supplementary Materials — With the manuscript, there are some supplementary materials. These supplementary figures and tables are quoted in the corresponding positions and can be viewed at any time. [file 5516563.f1.docx]

Table S1: Pesticides registered by 2020 for use on ginseng in China.

| **No.** | **Active ingredient** | **Class** | **Use** |
| --- | --- | --- | --- |
| 1 | Azoxystrobin^*^ | Fungicide | Control of black spot |
| 2 | *Bacillus subtilis* | Fungicide | Control of black spot, gray mold, damping-off |
| 3 | Carbendazim* | Fungicide | Control of rust rot |
| 4 | Cymoxanil* | Fungicide | Control of blight |
| 5 | Cyprodinil* | Fungicide | Control of gray mold |
| 6 | Copper hydroxide | Fungicide | Control of black spot |
| 7 | Copper oxychloride | Fungicide | Control of black spot |
| 8 | Diethofencarb* | Fungicide | Control of gray mold |
| 9 | Difenoconazole* | Fungicide | Control of black spot |
| 10 | Dimethomorph* | Fungicide | Control of blight |
| 11 | Fluazinam* | Fungicide | Control of blight |
| 12 | Fludioxonil* | Fungicide | Control of damping-off |
| 13 | Flumorph* | Fungicide | Control of blight |
| 14 | Fluopyram* | Fungicide | Control of gray mold |
| 15 | Flusilazole* | Fungicide | Control of powdery mildew |
| 16 | Gibberellic acid | Plant growth regulator | Increase germination rate; increase 1000-grain weight |
| 17 | Hymexazol | Fungicide | Control of root rot |
| 18 | Iprodione | Fungicide | Control of black spot, blight |
| 19 | Kresoxim-methyl* | Fungicide | Control of black spot |
| 20 | Mancozeb | Fungicide | Control of black spot |
| 21 | Mandipropamid* | Fungicide | Control of blight |
| 22 | Metalaxyl* | Fungicide | Control of blight |
| 23 | *Paenibacillus polymyza* | Fungicide | Control of damping-off |
| 24 | Polyoxin | Fungicide | Control of black spot |
| 25 | Propamocarb* | Fungicide | Control of blight |
| 26 | Propiconazole* | Fungicide | Control of black spot |
| 27 | Pyraoxystrobin* | Fungicide | Control of blight |
| 28 | Pyrimethanil* | Fungicide | Control of gray mold |
| 29 | Sodium dichloroisocyanurate | Fungicide | Control of damping-off |
| 30 | Thiamethoxam* | Insecticide | Control of wire worm |
| 31 | *Trichoderma harzianum* | Fungicide | Control of gray mold, damping-off |
| 32 | Trifloxystrobin* | Fungicide | Control of gray mold |

*, Pesticides involved in this method.

Table S2: Intra-day (n = 5) and inter-day (n = 15) recoveries, and the corresponding relative standard deviations (RSD) of 21 pesticides from ginseng using the developed method.

| **Compound** | **0.02 mg kg^−1^** | | | | **0.05 mg kg^−1^** | | | | **0.1 mg kg^−1^** | | | | **1 mg kg^−1^** | | | |
| --- | --- | --- | --- | --- | --- | --- | --- | --- | --- | --- | --- | --- | --- | --- | --- | --- |
|  | **Day 1^a^** | **Day 2^a^** | **Day 3^a^** | **InterD^a^** | **Day 1^a^** | **Day 2^a^** | **Day 3^a^** | **InterD^a^** | **Day 1^a^** | **Day 2^a^** | **Day 3^a^** | **InterD^a^** | **Day 1^a^** | **Day 2^a^** | **Day 3^a^** | **InterD^a^** |
| Azoxystrobin | 95(5) | 94(5) | 103(8) | 98(7) | 77(5) | 99(3) | 103(8) | 93(14) | 103(5) | 100(2) | 104(6) | 102(5) | 98(2) | 94(3) | 99(2) | 97(3) |
| Carbendazim | 78(14) | 93(7) | 93(8) | 88(12) | 73(11) | 95(9) | 91(6) | 86(14) | 84(4) | 90(6) | 82(7) | 85(7) | 84(8) | 82(3) | 79(2) | 82(5) |
| Cymoxanil | - | - | - | - | 108(9) | 72(16) | 79(16) | 87(22) | 112(16) | 102(15) | 89(14) | 101(17) | 105(10) | 81(8) | 86(4) | 90(14) |
| Cyprodinil | 82(11) | 85(10) | 102(7) | 90(14) | 80(7) | 99(9) | 95(5) | 91(11) | 93(4) | 96(7) | 100(4) | 96(6) | 93(2) | 91(4) | 90(2) | 91(3) |
| Diethofencarb | - | - | - | - | 81(13) | 108(6) | 119(1) | 101(18) | 98(13) | 99(14) | 110(11) | 103(13) | 105(5) | 102(5) | 100(11) | 102(7) |
| Difenoconazole | 99(15) | 100(8) | 96(8) | 98(10) | 88(6) | 109(11) | 98(4) | 98(12) | 94(7) | 105(2) | 104(4) | 101(7) | 95(6) | 100(5) | 92(6) | 95(6) |
| Dimethomorph | 96(8) | 94(7) | 106(4) | 99(8) | 76(2) | 98(3) | 91(3) | 88(11) | 103(3) | 100(3) | 105(3) | 103(4) | 87(1) | 92(2) | 97(5) | 92(6) |
| Fluazinam | - | - | - | - | 89(11) | 117(6) | 102(18) | 103(16) | 111(9) | 97(7) | 100(15) | 103(12) | 92(5) | 91(8) | 103(5) | 95(8) |
| Fludioxonil | - | - | - | - | 90(12) | 94(12) | 108(7) | 97(12) | 98(11) | 93(6) | 105(10) | 99(10) | 96(3) | 93(5) | 96(8) | 95(5) |
| Flumorph | 91(7) | 100(12) | 99(10) | 96(10) | 90(7) | 99(5) | 99(6) | 96(7) | 103(2) | 101(3) | 99(6) | 101(4) | 99(7) | 91(1) | 92(5) | 94(6) |
| Fluopyram | 95(7) | 95(5) | 100(7) | 96(6) | 75(9) | 101(4) | 118(4) | 98(20) | 96(3) | 92(3) | 105(6) | 98(7) | 100(4) | 99(8) | 92(7) | 97(7) |
| Flusilazole | 99(6) | 105(7) | 92(3) | 98(8) | 92(3) | 103(3) | 99(5) | 98(6) | 94(5) | 98(8) | 98(3) | 96(6) | 91(5) | 98(7) | 91(5) | 93(6) |
| Kresoxim-methyl | - | - | - | - | 83(9) | 100(18) | 119(11) | 99(20) | 99(10) | 102(13) | 91(12) | 97(12) | 89(10) | 102(10) | 93(7) | 95(10) |
| Mandipropamid | 93(7) | 84(10) | 108(8) | 95(13) | 84(8) | 103(8) | 104(6) | 97(12) | 93(5) | 97(3) | 108(3) | 99(7) | 97(2) | 93(4) | 90(4) | 93(4) |
| Metalaxyl | 91(11) | 99(3) | 104(11) | 98(10) | 85(9) | 95(4) | 100(2) | 94(9) | 93(5) | 92(7) | 99(6) | 95(7) | 94(3) | 90(4) | 95(2) | 93(4) |
| Propamocarb | - | - | - | - | 74(8) | 89(5) | 93(4) | 85(12) | 81(5) | 85(5) | 90(2) | 85(6) | 78(8) | 78(3) | 77(4) | 77(5) |
| Propiconazole | 95(11) | 95(5) | 112(6) | 101(11) | 83(9) | 103(7) | 98(12) | 95(13) | 90(11) | 107(4) | 105(6) | 101(10) | 98(5) | 94(4) | 94(5) | 95(5) |
| Pyraoxystrobin | 84(5) | 89(9) | 96(4) | 90(8) | 79(3) | 97(6) | 97(9) | 91(12) | 92(3) | 98(13) | 101(4) | 97(9) | 96(2) | 95(3) | 95(6) | 95(4) |
| Pyrimethanil | 86(7) | 97(6) | 107(6) | 97(11) | 80(9) | 98(9) | 97(8) | 91(12) | 96(4) | 93(6) | 91(6) | 93(5) | 87(3) | 92(6) | 90(3) | 89(5) |
| Thiamethoxam | - | - | - | - | 79(15) | 104(15) | 102(16) | 95(19) | 84(8) | 95(12) | 108(12) | 96(15) | 91(5) | 91(2) | 85(5) | 89(5) |
| Trifloxystrobin | 90(7) | 107(19) | 92(6) | 96(14) | 76(10) | 90(13) | 102(10) | 90(16) | 102(5) | 88(11) | 105(11) | 99(12) | 90(6) | 93(6) | 103(5) | 95(8) |

^a^, recovery/%(RSD/%).


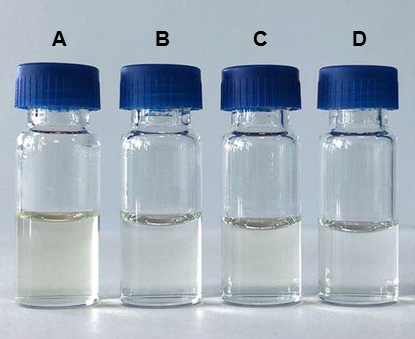


Figure S1: Ginseng sample extract (A) without clean-up, (B) purified by PSA + C_18_, (C) purified by PSA + FLS, (D) purified by PSA + Z-Sep.


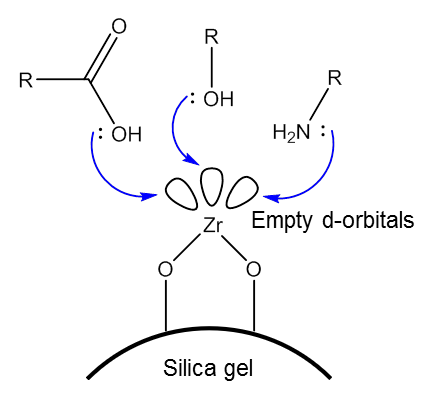


Figure S2: Schematic diagram of the matrix removal mechanism of Z-Sep.


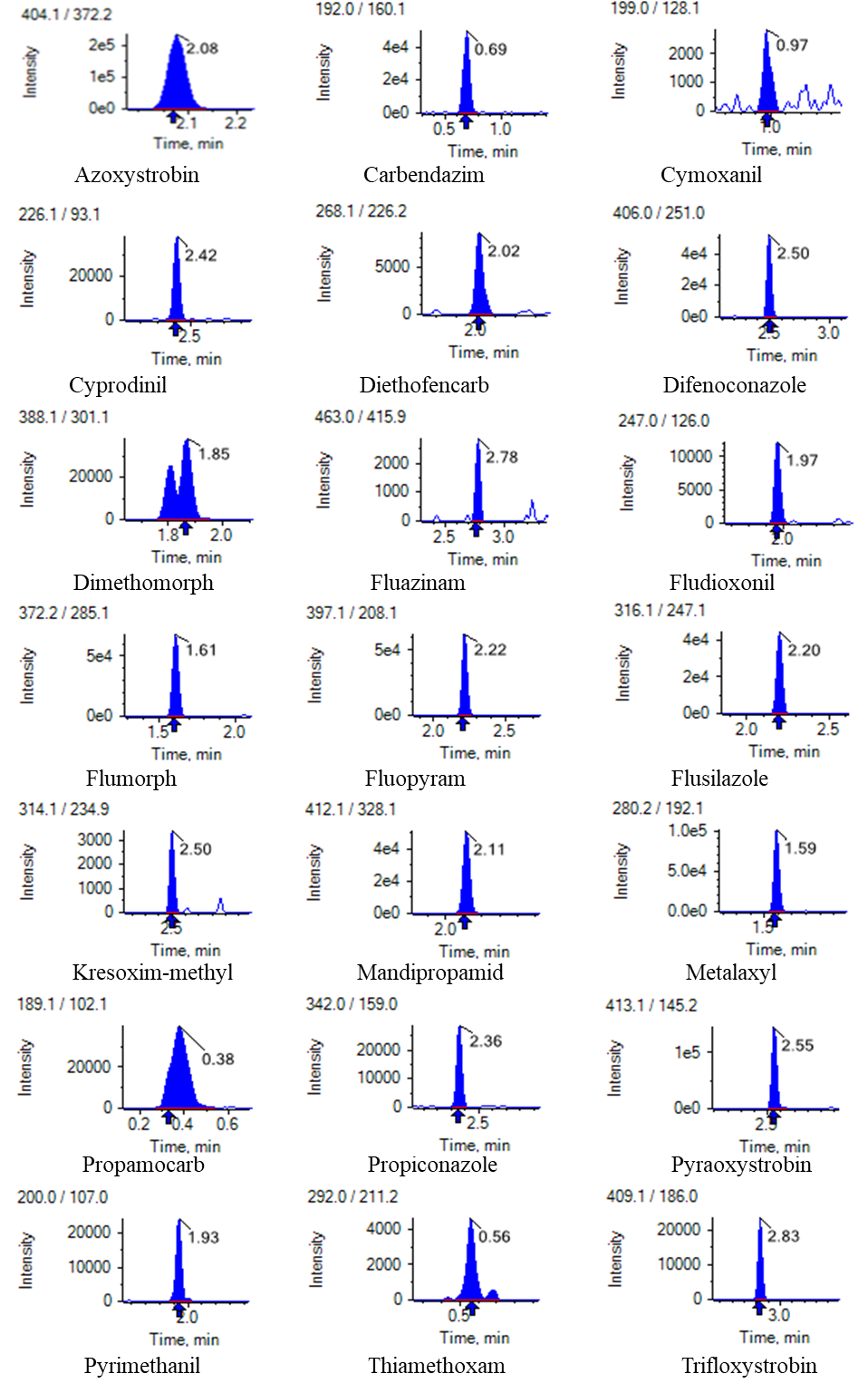


Figure S3: Typical MRM chromatograms of 21 pesticides in ginseng spiked at LOQs.
